# CXCL8 expression is associated with advanced stage, right sidedness, and distinct histological features of colorectal cancer

**DOI:** 10.1002/cjp2.290

**Published:** 2022-07-25

**Authors:** Kathryn AF Pennel, Jean A Quinn, Colin Nixon, Jitwadee Inthagard, Hester C van Wyk, David Chang, Selma Rebus, GPOL Group, Jennifer Hay, Noori N Maka, Campbell SD Roxburgh, Paul G Horgan, Donald C McMillan, James H Park, Antonia K Roseweir, Colin W Steele, Joanne Edwards

**Affiliations:** ^1^ Wolfson Wohl Cancer Research Institute, Institute of Cancer Sciences University of Glasgow Glasgow UK; ^2^ CRUK Beatson Institute Glasgow UK; ^3^ Department of Surgery University of Glasgow, Glasgow Royal Infirmary Glasgow UK; ^4^ Glasgow Precision Oncology Laboratory, Wolfson Wohl Cancer Research Centre, Institute of Cancer Sciences University of Glasgow Glasgow UK; ^5^ Glasgow Tissue Research Facility Queen Elizabeth University Hospital Glasgow UK; ^6^ Department of Pathology Queen Elizabeth University Hospital Glasgow UK; ^7^ Department of Surgery Queen Elizabeth University Hospital Glasgow UK; ^8^ School of Medicine, Dentistry and Nursing University of Glasgow Glasgow UK

**Keywords:** *CXCL8*, CXCR2, colorectal cancer, sidedness, stromal invasion, tumour microenvironment, metastases

## Abstract

CXCL8 is an inflammatory chemokine elevated in the colorectal cancer (CRC) tumour microenvironment. CXCR2, the major receptor for CXCL8, is predominantly expressed by neutrophils. In the cancer setting, CXCL8 plays important roles in neutrophil chemotaxis, facilitating angiogenesis, invasion, and metastasis. This study aimed to assess the spatial distribution of *CXCL8* mRNA expression in CRC specimens, explore associations with clinical characteristics, and investigate the underlying biology of aberrant *CXCL8* levels. CXCR2 expression was also assessed in a second cohort of unique CRC primary tumours and synchronously resected matched liver metastases. A previously constructed tissue microarray consisting of a cohort of stage I–IV CRC patients undergoing surgical resection with curative intent (*n* = 438) was probed for *CXCL8* via RNAscope®. Analysis was performed using HALO® digital pathology software to quantify expression in the tumour and stromal compartments. Scores were assessed for association with clinical characteristics. Mutational analyses were performed on a subset of these patients to determine genomic differences in patients with high *CXCL8* expression. A second cohort of stage IV CRC patients with primary and matched metastatic liver tumours was stained via immunohistochemistry for CXCR2, and scores were assessed for clinical significance. *CXCL8* expression within the stromal compartment was associated with reduced cancer‐specific survival in the first cohort (*p* = 0.035), and this relationship was potentiated in right‐sided colon cancer cases (*p* = 0.009). High *CXCL8* within the stroma was associated with driving a more stromal‐rich phenotype and the presence of metastases. When stromal *CXCL8* scores were combined with tumour‐infiltrating macrophage counts or systemic neutrophil counts, patients classified as high for both markers had significantly poorer prognosis. CXCR2+ immune cell infiltration was associated with increased stromal invasion in liver metastases (*p* = 0.037). These data indicate a role for *CXCL8* in driving unfavourable tumour histological features and promoting metastases. This study suggests that inhibiting *CXCL8*/CXCR2 should be investigated in patients with right‐sided colonic disease and stroma‐rich tumours.

## Introduction

Colorectal cancer (CRC) remains a leading cause of worldwide cancer‐related mortality. To improve survival outcomes, novel biomarkers and therapeutic targets need to be identified. Over the past decade, treatment of certain cancer types has been revolutionised by the adoption of immune checkpoint blockade; however, this is only utilised successfully in a subset of CRC patients with advanced‐stage mismatch repair deficient (dMMR) disease [1]. The observed variety in response to these targeted immunotherapies is attributed to the immunosuppressive nature of microsatellite stable (MSS) tumours and heterogeneity associated with CRC. MSS tumours account for ~85% of CRC and are characterised by lower immune infiltration, immune exclusion, and decreased presence of neoantigens compared to dMMR tumours [2, 3]. These MSS tumours generally have a lower tumour mutational burden which results in decreased major histocompatibility complex expression on the surface of antigen‐presenting cells, further exacerbating the immunosuppressive nature of MSS disease [2, 3].

Tumour heterogeneity can be driven by dysregulation of many different cellular signalling networks leading to promotion of the hallmarks of cancer [4]. Aberrant cell signalling is initiated via the overexpression of specific cytokines and chemokines produced by many different cell types in the tumour microenvironment (TME). Targeting this dysregulation represents a promising therapeutic strategy for novel and repurposed drugs to be utilised in combination with standard‐of‐care chemotherapy in CRC patients [5].

C‐X‐C motif chemokine legend 8 (*CXCL8*) is a signalling molecule elevated in the cancer setting both systemically and within the TME of several solid tumour types [6, 7, 8]. *CXCL8* functions as a neutrophil chemoattractant via their surface expression of CXCR2 [9]. When *CXCL8* binds to CXCR2 signal, transduction results in the promotion of angiogenesis, cell survival, migration, proliferation, and invasion [9]. Downstream transcription factors and pathways which CXCL8 activates include mitogen‐activated protein kinase (MAPK), protein kinase B (Akt), extracellular‐signal‐regulated kinase (ERK), and signal transducer and activator of transcription 3 (STAT3), all of which have been linked to tumour progression and hyperactivity is associated with poor clinical outcomes [10, 11].

Although a prognostic role for *CXCL8* in CRC already exists in the literature, there is limited evidence regarding the importance of the spatial distribution of *CXCL8* expression within different compartments of the TME. Production of *CXCL8* in the TME can be influenced by the inflammatory milieu including the presence of CXCL12, IL‐1, IL‐6, TNF‐α, and factors such as hypoxia and reactive oxygen species [12]. Numerous cell types can produce *CXCL8* including epithelial cells, endothelial cells, tumour‐associated macrophages, cancer‐associated fibroblasts (CAFs), and tumour cells themselves [11]. Therefore, the spatial distribution of *CXCL8* expression in the TME could be important in furthering our understanding of the biology of *CXCL8* in driving cancer progression.

This study aimed to investigate the prognostic role of *CXCL8* within the tumour epithelium and tumour‐associated stroma utilising a retrospective cohort of stage I–IV CRC patients undergoing surgery with curative intent. Expression of *CXCL8* mRNA within each TME compartment (tumour epithelium/tumour‐associated stroma) was assessed for association with clinical characteristics including survival outcomes and tumour histology. Furthermore, the underlying biology of patients with high levels of *CXCL8* was investigated using matched mutational data. A second retrospective cohort of synchronously resected primary tumours and matched liver metastases was assessed via immunohistochemistry (IHC) for protein expression of the *CXCL8* receptor CXCR2 to determine any association with clinicopathological features in the metastatic setting.

## Materials and methods

### Patient cohorts

A retrospective cohort (cohort 1) consisting of 1,030 stage I–IV CRC patients undergoing potentially curative resection across Greater Glasgow and Clyde (GGC) hospitals between 1997 and 2007 was utilised in the study. Tumours were staged with the fifth edition of TNM staging and clinical follow‐up data were last updated in 2017 from NHS GGC Safe Haven data. At this time, 324 patients (32%) had died of primary CRC, 332 patients (32.8%) had died of other causes, and 355 patients (35.1%) were still alive. Cancer‐specific survival (CSS) (date of surgery until the last follow‐up) was used as a clinical endpoint throughout this study. Mean follow‐up time was 139 months. Patients were excluded from the analysis if they received neoadjuvant therapy, emergency surgery, and/or died within 30 days of surgical procedure. Due to the limited tissue left in the blocks of each tissue microarray (TMA), valid cores were only available for 438 patients from the cohort. This study was approved by the West of Scotland Research Ethics Committee (16/WS/0207) and patient information is held within the Glasgow and Clyde Safe Haven (12/WS/0142).

A second cohort (cohort 2) consisted of 46 stage IV CRC patients who underwent synchronous resection of colorectal primary tumour and liver metastases between April 2002 and June 2010 at Glasgow Royal Infirmary. Information on date and cause of death was determined via access to the NHS GGC clinical portal. Clinical follow‐up data were last updated in 2017 and at this time the mean survival time was 40.14 months and 40% of patients (*n* = 24) were alive, 50% (*n* = 30) had died of cancer, and 5% (*n* = 3) had died of unrelated causes. Due to the size of cohort 2, no exclusion criteria were applied prior to statistical analysis. This study was approved by the West of Scotland Research Ethics Committee (#357).

### RNAscope®

RNA *in situ* hybridisation using RNAscope (ACD Bio, Newark, CA, USA) was performed at the CRUK Beatson Institute (performed by CN) on previously constructed TMAs consisting of patients from cohort 1 to detect the *PPIB* housekeeping gene and *CXCL8* mRNA. Staining was performed using a Leica Bond Rx system (Leica Biosystems, Wetzlar, Germany). Expression was quantified using Halo digital pathology software (Indica Labs, Albuquerque, NM, USA) in copies per μm^2^. A classifier was built to distinguish between tumour epithelium and stromal‐rich areas of the TMA cores. Raw scores for *CXCL8* expression within the tumour and stroma were normalised to *PPIB* scores. Cut‐offs for high and low expression were determined using survminer, survival, maxstat, and tidyverse packages in R studio (v1.3) based on CSS (RStudio, Boston, MA, USA).

A subset of the cohort (*n* = 12) was dual stained/probed for alpha‐smooth muscle actin (α‐SMA) protein and *CXCL8* RNA to confirm the presence of *CXCL8* mRNA within the stroma. Co‐localisation staining was performed on 4‐μm formalin‐fixed paraffin‐embedded sections (FFPE) full sections which had previously been baked at 60 °C for 2 h. The staining was performed on a Leica Bond Rx strictly following Bio‐Techne's co‐localisation kit/protocol. The RNAscope probe used was Hs‐IL8 (310388, Bio‐Techne, Minneapolis, MN, USA) and the α‐SMA antibody clone D4K9N (19245, Cell Signaling Technologies, Boston, MA, USA).

### Immunohistochemistry

Immunohistochemical staining was performed on FFPE resections from cohort 2 to assess the expression of CXCR2 on immune cells in the TME. Staining was performed using the Leica Bond Rx autostainer. Sections underwent on‐board dewaxing (AR9222, Leica) followed by heat‐induced epitope retrieval using ER2 retrieval buffer (AR9640, Leica). The sections were stained with CXCR2 primary antibody (PA1‐20673, Thermo Fisher Scientific, Walton, MA, USA) at a dilution of 1:100 followed by rabbit envision secondary antibody (K4003, Agilent Technologies, Santa Clara, CA, USA). Sections were visualised using diaminobenzidine before counterstaining with haematoxylin using an Intense R Kit (DS9263, Leica Biosystems). Stained sections were scanned onto the Slidepath platform (Leica Biosystems, Milton Keynes, UK) using a Hamamatsu NanoZoomer (Hamamatsu, Welwyn Garden City, UK) for visualisation. Assessment of immune cell infiltration was performed at ×20 objective magnification using a point score digital algorithm available within the Slidepath platform, validated by 10% non‐automated point counts of the same area (performed by KAFP). Cells were counted in three different locations of each tumour within a 4‐μm^2^ grid, and an average taken to account for heterogeneity. Only fields within the tumour (including cancer cell nests and surrounding tissue stroma) were counted. Scores were averaged and median values were utilised as a cut‐off for high and low expression.

### Histopathological phenotype assessments

Tumour stroma percentage (TSP) assessment was performed as previously described [13]. In brief, full face haematoxylin and eosin‐stained sections were assessed manually for the composition of stromal cells within the intra‐tumour area, performed by JHP and AKR with validation by JE and further validation on a subset of the cohort performed by a clinical pathologist (NNM). Tumours with >50% stromal volume were graded as high, and ≤50% was considered low. Klintrup–Makinen (KM) grade was determined as previously described [14]. The invasive margin was analysed for the presence of immune cells, patients with a florid cup (3) or thin continuous band of cells (2) were considered high for immune influx. Tumours with a patchy band (1) or no immune cells present at the margin were considered low for immune infiltration.

### Mutational profiling

Mutational profiling was performed on a subset of patients from cohort 1 (*n* = 252). DNA was previously extracted from FFPE sections by NHS molecular diagnostics, Dundee. DNA quality and concentration were determined using the Qubit assay (Thermo Fisher). Sequencing was outsourced and performed by the Glasgow Precision Oncology Laboratory (GPOL) using a custom in‐house designed panel of 151 cancer‐associated genes run on a HiSeq4000 machine (Illumina, San Diego, CA, USA).

The publicly available cBioPortal resource was utilised to validate findings in the Cancer Genome Atlas program (TCGA)/PanCancer Atlas adenocarcinoma cohort (*n* = 594) available at https://www.cbioportal.org/.

### Statistical analyses

Data analysis of *CXCL8* expression in cohort 1 was performed using IBM SPSS (Chicago, IL, USA). The relationship between tumour cell/stromal *CXCL8* expression and CSS was assessed using Kaplan–Meier survival curves. Chi‐squared tests were utilised to assess associations between *CXCL8* expression and clinicopathological features. Analysis of mutational profiling data was performed using Maftools in R Studio (v1.3, RStudio, PBC, Boston, MA, USA). Oncoplots and a forest plot were constructed to visualise differences in mutations between high and low stromal *CXCL8* groups. Statistical significance was set to *p* < 0.05.

## Results

### Expression of *CXCL8* within the stromal compartment is associated with reduced CSS

**Figure 1:**
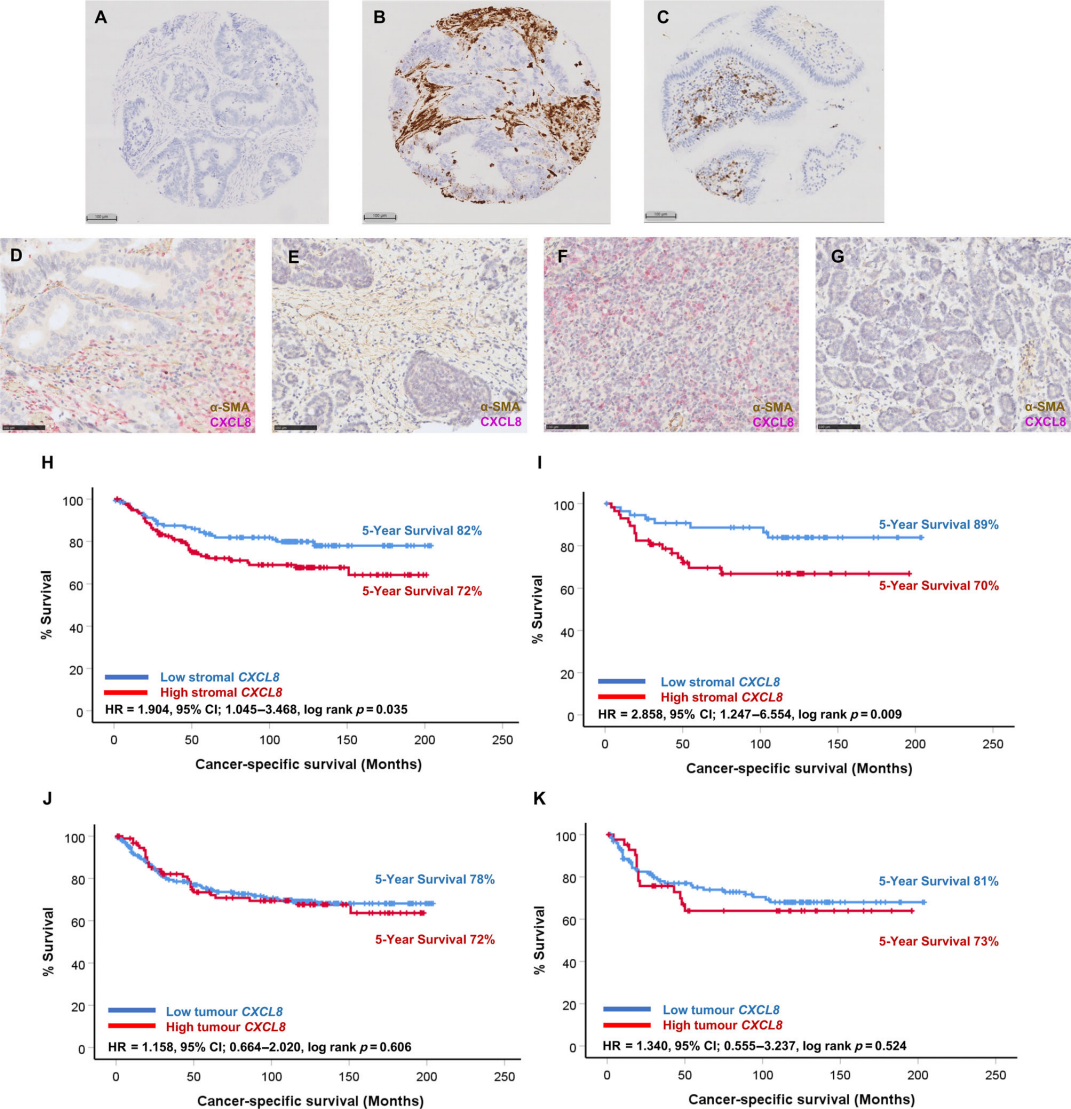
High *CXCL8* expression in the stromal compartment is associated with prognosis in the full Glasgow combined cohort and this is potentiated in right‐sided colonic disease. (A–C) Representative images of negative, high stromal, and high tumour cell *CXCL8* expression in TMAs from cohort 1 probed by RNAscope®. (D–G) Representative images showing dual‐stained tumour sections probed for *CXCL8* and stained immunohistochemically for α‐SMA. Kaplan–Meier survival analysis of stromal *CXCL8* expression in (H) the full cohort and (I) in right‐sided colon cases only. Kaplan–Meier survival analysis of tumour *CXCL8* expression in (J) the full cohort and (K) in right‐sided colon cases.

After exclusion criteria were applied to the CRC cohort of 438 patients from cohort 1, 380 patients had valid *CXCL8* scores and were included in downstream analyses (supplementary material, Figure S1). Representative images of TMA cores negative, low, and high for *CXCL8* expression are shown in Figure 1A–C. Positive staining was detected within the tumour and stromal compartments and full sections from a subset of patients were dual stained/probed for α‐SMA via IHC and *CXCL8*, with representative images of high/low expression in α‐SMA‐positive and ‐negative areas shown in Figure 1D–G. The mean score for stromal *CXCL8* was 0.79 copies per μm^2^ (*n* = 386) and for *CXCL8* within tumour cells was 0.58 copies per μm^2^ (*n* = 387). Survminer cut‐off point analysis using the continuous variables determined the optimal cut‐off point for high and low expression to be 0.32 copies per μm^2^ for stromal *CXCL8* and 0.65 copies per μm^2^ for tumour cell *CXCL8*. This resulted in 194 patients classified as high for stromal *CXCL8* and 192 classified as low. For tumour cell *CXCL8* expression, 95 patients were classified as high and 290 fell into the low group. Pearson correlation analysis revealed a weakly positive correlation between *CXCL8* expression counts from the stroma and tumour cell compartments (*r* = 0.224, *p* < 0.001).

Kaplan–Meier survival analysis identified a significant association between high *CXCL8* mRNA expression within the stromal compartment and reduced CSS in the full cohort (HR = 1.904, 95% CI: 1.045–3.468, log‐rank *p* = 0.035) (Figure 1H). Patients classified as high for stromal *CXCL8* (*n* = 133) had a mean survival time of 148 (95% CI: 133–162) months compared to 169 (95% CI: 157–181) months observed in patients classified as low for stromal *CXCL8* expression (*n* = 137). This relationship was potentiated in patients with right‐sided colon disease (HR = 2.858, 95% CI: 1.247–6.554, log‐rank *p* = 0.009) (Figure 1I). In right‐sided cases, patients classified as high for stromal *CXCL8* (*n* = 56) observed a mean survival time of 140 (95% CI: 118–162) months compared with 181 (95% CI: 166–197) months in patients classified as low for expression of stromal *CXCL8* (*n* = 56). When cases were stratified by MMR status, there was a significant association between stromal *CXCL8* expression and CSS in pMMR (*p* = 0.021) but not dMMR cases (*p* = 0.565); however, this may be due to limited patient numbers in the dMMR group (supplementary material, Figure S2). Kaplan–Meier survival analyses showed that there was no association between *CXCL8* expression within the tumour cell compartment and CSS in the full cohort (Figure 1J) or when the cohort was stratified to include only right‐sided tumours (Figure 1K). Stromal *CXCL8* expression was associated with outcome at the univariate level (*p* = 0.038), but was not found to be independently prognostic upon multivariate survival analysis of the full cohort (*p* = 0.071) (supplementary material, Table S1).

### High stromal *CXCL8* expression is associated with unfavourable tumour histological features

**Figure 2:**
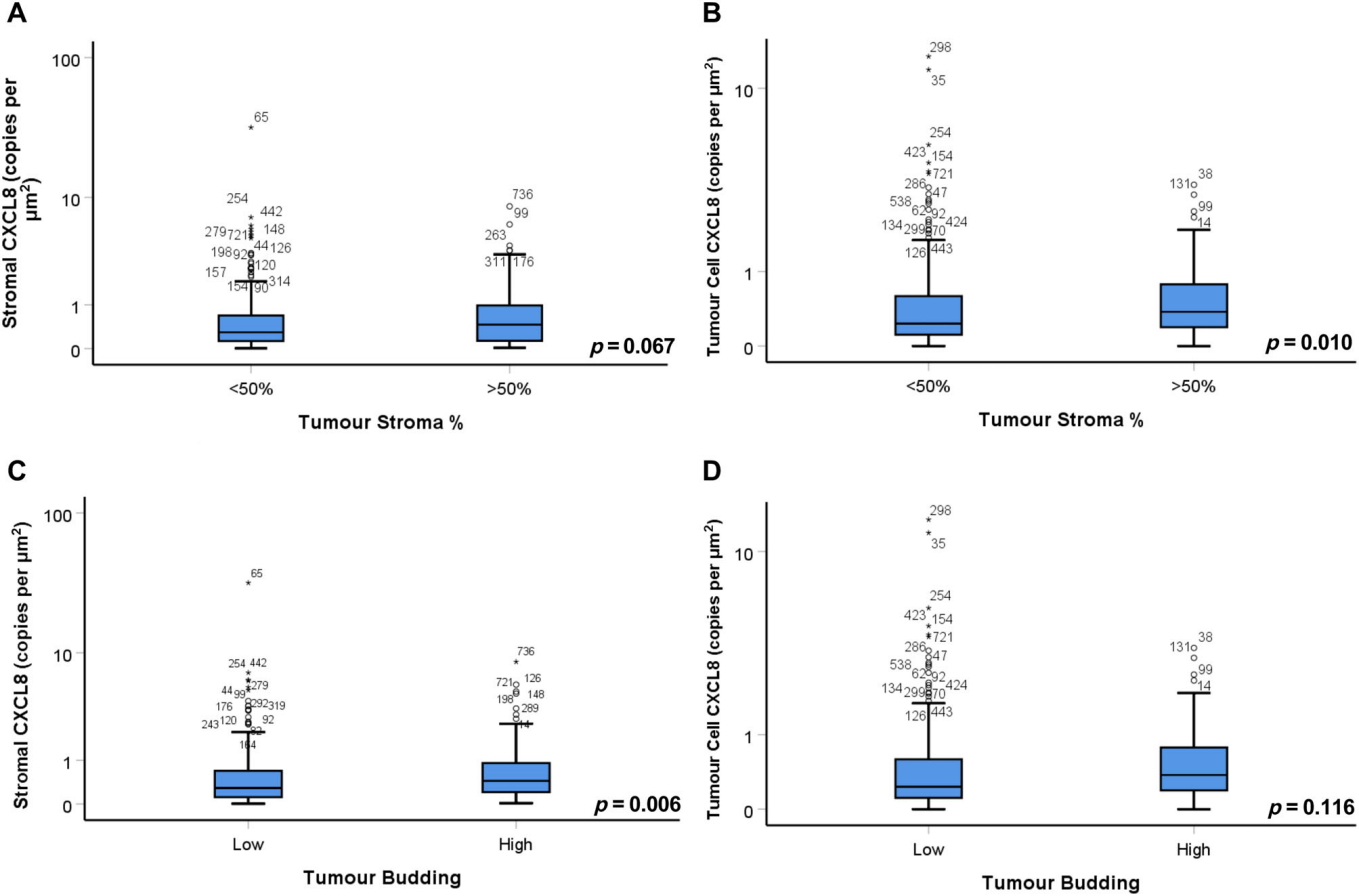
*CXCL8* expression is elevated in stroma‐rich tumours. Box plots showing the level of stromal and tumour *CXCL8* mRNA expression relative to (A and B) TSP and (C and D) tumour budding with significance assessed via Kruskal–Wallis non‐parametric tests.

Chi‐squared tests were performed to determine any association between stromal *CXCL8* expression and clinical characteristics/histological tumour features (Table 1). High *CXCL8* within the stromal compartment was significantly associated with a higher TSP (*p* = 0.040) and higher frequency of tumour budding (*p* = 0.002). There was a significant association between high stromal *CXCL8* and Ki67 proliferation index, with the middle quartiles of Ki67 enriched for *CXCL8* expression. This steady‐state level of tumour proliferation generally confers worse prognosis due to the provision of optimal conditions for angiogenesis and hypoxia. At higher levels of proliferation, the tumour can outgrow the blood supply and become necrotic, and lower levels of proliferation generally ameliorate the anti‐tumour immune response. In stromally dense tumours (>50% TSP), there was a significant elevation in tumour *CXCL8* expression (*p* = 0.010) and a trend towards higher stromal *CXCL8* (*p* = 0.067) when assessed via non‐parametric Kruskal–Wallis *H* tests (Figure 2A,B). The high frequency of tumour buds was associated with increased stromal *CXCL8* expression (*p* = 0.010) and to a lesser extent tumour *CXCL8* mRNA copies (*p* = 0.116) (Figure 2C,D).

**Table 1:** Association between stromal *CXCL8* and clinicopathological characteristics

	Stromal *CXCL8* expression		
Clinical characteristic	Low (*n* = 140)	High (*n* = 136)	*p*
Age			
≤65	36 (25.7)	35 (25.7)	0.533
>65	104 (74.3)	101 (74.3)	
Sex			
Male	66 (47.1)	72 (52.9)	0.200
Female	74 (52.9)	64 (47.1)	
T stage			
1	10 (7.1)	4 (2.9)	0.107
2	35 (25.0)	23 (16.9)	
3	67 (47.9)	78 (57.4)	
4	28 (20.0)	31 (22.8)	
N stage			
0	100 (71.4)	84 (61.8)	0.226
1	29 (20.7)	39 (28.7)	
2	11 (7.9)	13 (9.6)	
M stage			
0	139 (100)	131 (96.3)	**0.028**
1	0 (0)	5 (3.7)	
Glasgow Microenvironment Score			
Immune	54 (38.8)	45 (33.1)	**0.044**
Intermediate	72 (51.8)	64 (47.1)	
Stromal	13 (9.4)	27 (19.9)	
TSP			
Low	117 (83.6)	101 (74.3)	**0.040**
High	23 (16.4)	35 (25.7)	
KM grade			
Low (0–1)	84 (60.4)	91 (66.9)	0.161
High (2–3)	55 (39.6)	45 (33.1)	
Ki67 proliferation index			
Low (≤30%)	39 (28.10)	59 (43.4)	**0.006**
High (>30%)	100 (71.9)	77 (56.6)	
Tumour budding			
Low	113 (80.7)	88 (64.7)	**0.002**
High	27 (19.3)	48 (35.3)	
MMR status			
MMR proficient	115 (82.7)	110 (80.9)	**0.404**
MMR deficient	24 (17.3)	26 (19.1)	

Chi‐squared table of associations between expression of *CXCL8* in the tumour‐associated stroma and clinical information/prognostic markers of tumour histology. Significant *P* values are given in bold font.

### Patients with high stromal *CXCL8* expression combined with high myeloid cell counts observe worse outcomes

**Figure 3:**
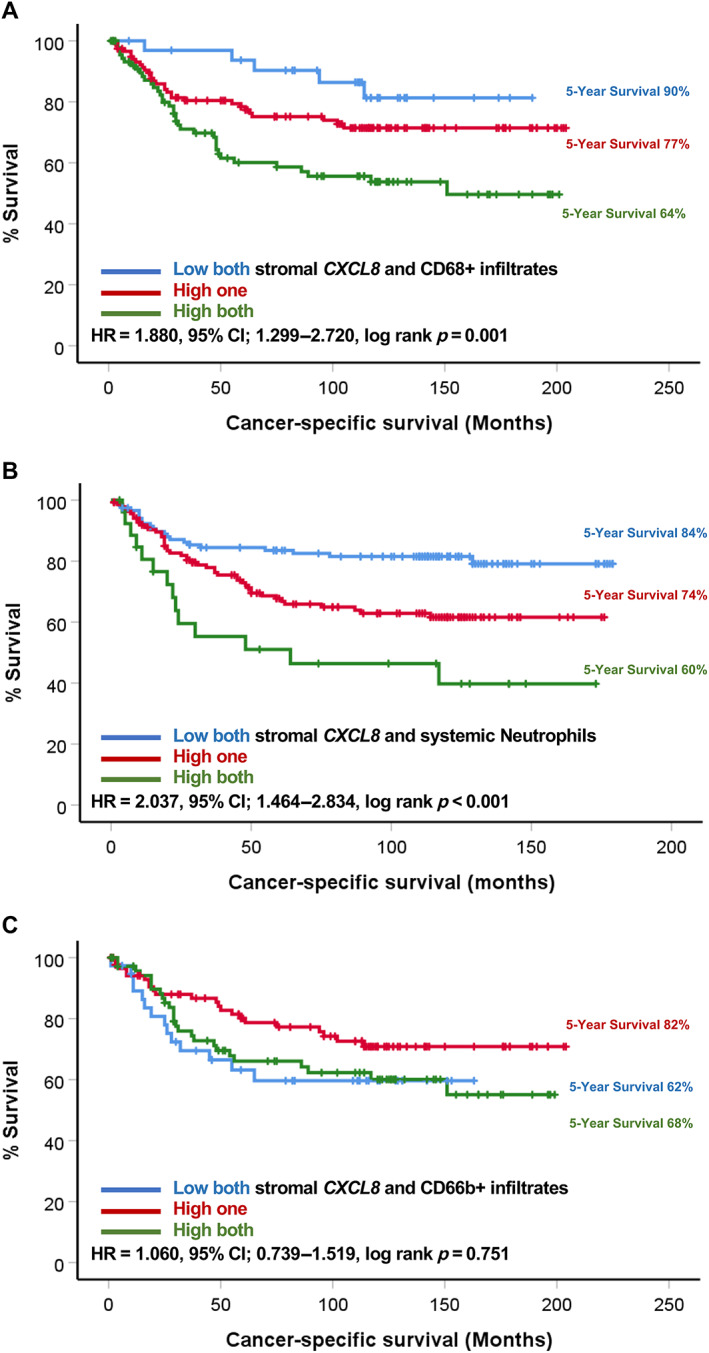
Myeloid cells and stromal *CXCL8* confer poor prognosis. Kaplan–Meier survival curves showing association between combined scores of (A) stromal *CXCL8* and CD68+ tumour‐infiltrating cells, (B) stromal *CXCL8* and systemic neutrophil counts, and (C) stromal *CXCL8* and CD66b+ tumour‐infiltrating cells.

Given the prominent role of *CXCL8* in neutrophil recruitment, stromal *CXCL8* expression was investigated in combination with myeloid cell infiltrates (CD66b+ cells and CD68+ cells) and systemic neutrophil counts. Kaplan–Meier survival analysis revealed a significant association between stromal *CXCL8* and CD68+ cell density, with a significant reduction in survival of patients whose tumours were high for both compared to those high for one and low for both markers (HR = 1.880, 95% CI: 1.299–2.720, *p* = 0.001) (Figure 3A). The mean survival time for patients with tumours low for both stromal *CXCL8* and CD68+ infiltration was 167 (95% CI: 150–184) months compared to 156 (95% CI: 141–170) months for one high and 122 (95% CI: 104–141) months for both low. Similarly, patients with high tumour stromal *CXCL8* and high systemic neutrophil counts observed worse outcomes than those high for one or low for both (HR = 2.037, 95% CI: 1.464–2.834, *p* < 0.001) (Figure 3B). Patients with high systemic neutrophil counts and high tumour stromal *CXCL8* expression observed a mean survival time of 150 (95% CI: 138–161) months, compared with 122 (95% CI: 110–135) months for one high and 88 (95% CI: 59–118) months in patients low for both markers. There was no significant association between combined stromal *CXCL8* and CD66b+ infiltrates and CSS (Figure 3C).

### Expression of stromal *CXCL8* is associated with a distinct mutational background

**Figure 4:**
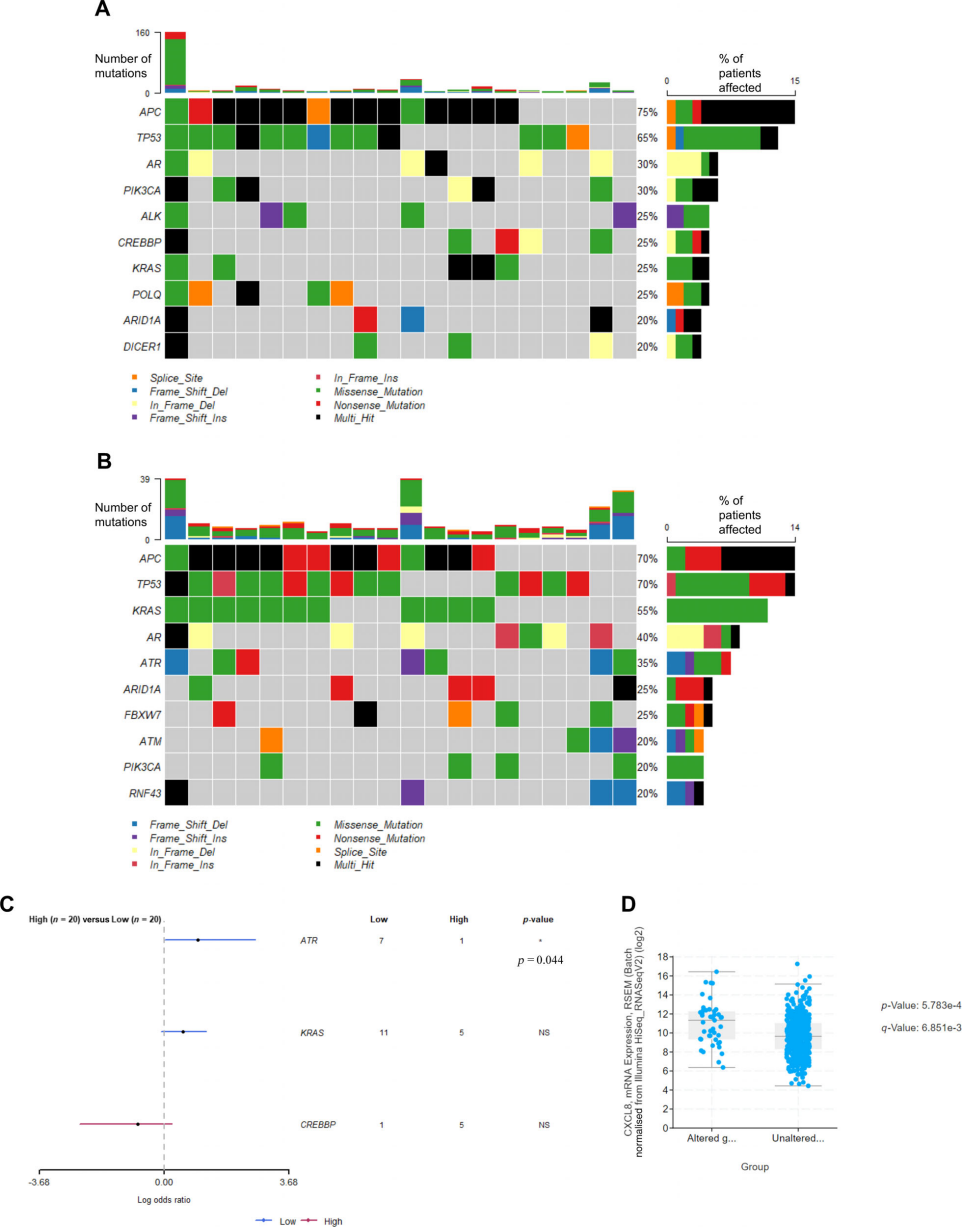
Expression of *CXCL8* in the stroma is associated with a distinct mutational background. Oncoplot showing the top 10 mutations identified in patients with (A) the highest (*n* = 20) and (B) the lowest (*n* = 20) expression of stromal *CXCL8*. (C) Forest plot highlighting the significantly differentially mutated genes between high and low *CXCL8* stromal groups. (D) Box plots showing the expression of *CXCL8* mRNA in the TCGA dataset in patients with and without alteration in the *CREBBP* gene (*n* = 526).

When the mutational background of patients from cohort 1 with the top 20 highest expression levels of stromal *CXCL8* was analysed, the most frequently mutated gene was *APC* (75% of cases), followed by *TP53* (65%) (Figure 4A). In terms of patients with the lowest tumour expression of stromal *CXCL8*, *APC* and *TP53* were mutated in 70% of cases (Figure 4B). *KRAS* was mutated in 55% of cases (Figure 4B). When Fishers' exact tests were performed to determine any differentially mutated genes between groups, DNA damage signalling kinase *ATR* (ataxia‐telangiectasia‐ and Rad3‐related) was significantly more likely to be mutated in the low stromal *CXCL8* group (*p* = 0.044) (Figure 4C). CREB‐binding protein (*CREBBP*) was mutated more frequently in tumours with high stromal *CXCL8* and when further analysis was performed on the TCGA/PanCancer Atlas dataset (*n* = 594) there was a significant association between *CREBBP* mutation and higher *CXCL8* mRNA expression (*p* < 0.001) (Figure 4D).

### CXCR2 expression is associated with increased stromal invasion in the metastatic setting

**Figure 5:**
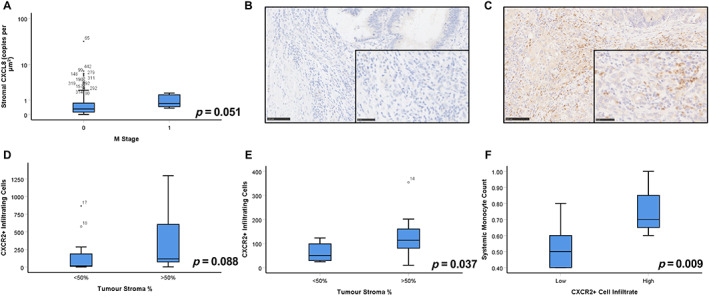
*CXCL8* is elevated in stage IV disease and expression of CXCR2 is associated with dense stromal invasion in primary CRC tumours and matched liver metastases. (A) Box plots showing the level of stromal *CXCL8* mRNA expression relative to absence or presence of metastases with significance assessed via Kruskal–Wallis tests. Representative images of (B) negative and (C) positive immunohistochemical staining of cohort 2 for CXCR2. (D and E) Box plots showing the level of CXCR2+ cell infiltration relative to TSP in (D) primary and (E) matched metastatic liver tumours. (F) Box plots showing the systemic monocyte counts relative to CXCR2+ infiltrates within the primary CRC tumours of cohort 2.

In cohort 1, expression of stromal *CXCL8* was significantly higher in patients with metastatic disease (Figure 5A). Therefore, a unique cohort of synchronously resected primary colorectal tumours and matched liver metastases (cohort 2) was stained for the main cognate receptor of *CXCL8*, CXCR2, by IHC. Positive staining was identified amongst the inflammatory infiltrate of some tumours, as shown in representative images (Figure 5B,C). The number of CXCR2+ immune cells (mainly neutrophils) at the invasive edge of the primary tumour significantly correlated with CXCR2+ infiltrates at the margin of the matched liver metastases (rho = 0.612, *p* = 0.003) (Figure 5D). In cohort 2, there was a trend towards increased infiltration of CXCR2+ cells in patients with high stromal invasion in primary tumours (*p* = 0.088) and a significant increase in matched liver metastases (*p* = 0.037) (Figure 5E,F). Representative images of stroma‐rich primary and metastatic tumours are shown in supplementary material, Figure S3.

## Discussion

This study has strongly implicated *CXCL8* mRNA expression within the tumour‐associated stroma as a marker of poor prognosis in CRC. Data from the literature corroborate these findings. A large meta‐analysis investigating both CXCL8 protein and RNA within the tissue and serum of over 1,500 CRC patients identified a strong association between high expression and poor clinical outcome [15]. The current study highlights the importance of the spatial distribution of *CXCL8* expression within the TME, as stromal but not tumour cell *CXCL8* expression was significantly prognostic. Furthermore, the findings from this study suggest that the prognostic influence of stromal *CXCL8* was potentiated in patients with right‐sided colonic tumours. Sidedness is an important clinical characteristic, with these patients more likely to have an elevated systemic inflammatory response and worse outcome [16, 17]. There is evidence that the inflammatory component of the TME is different in right‐sided tumours compared to other disease sites. The potentiation of the prognostic nature of *CXCL8* observed in this study is likely attributed to an immune‐related effect. Previous studies have shown that right‐sided tumours have an increased influx of immune cells, particularly CD8+ T cells, and higher expression of PD‐L1 on the surface of tumour cells [18, 19]. In a study of urethral and renal cell carcinomas, both circulating CXCL8 protein and *CXCL8* RNA were elevated in the peripheral blood monocular cells of patients who did not respond to PD‐1 checkpoint inhibitors [20]. We hypothesise that one mechanism of tumour promotion induced by *CXCL8* is by its augmentation of the expression of PD‐L1 on tumour cells. Therefore, inhibition of *CXCL8* in combination with checkpoint inhibitors represents an interesting approach which merits investigation in preclinical studies.

In terms of the mechanisms of tumour promotion, there is evidence that secretion of *CXCL8* promotes many of the hallmarks of cancer. *In vitro* studies of pancreatic cancer have shown that *CXCL8* works synergistically with CXCL12 to promote angiogenesis and invasion [21]. Similarly, in prostate cancer cell lines, *CXCL8* contributed to increased proliferation and invasion [22]. In the present study, high expression of *CXCL8* within the stroma was significantly associated with adverse histological features including high presence of tumour buds, stromal invasion, and moderate Ki67 proliferation index. Stroma‐rich, high‐budding phenotypes confer poor prognosis and further work is required to elucidate if inhibition of *CXCL8* or associated receptor/s could dampen stromal recruitment and invasion [13, 21, 23]. Previous data from gastric cancer models have shown that *CXCL8* specifically derived from CAFs was implicated in driving resistance to chemotherapy [24]. In this study, combined scores of stromal *CXCL8* and tumour‐infiltrating macrophages (CD68+ cells) or systemic neutrophil counts significantly stratified patient survival. Patients high for both markers had reduced CSS compared to those low for one or both markers. Interestingly, there was no association between combined stromal *CXCL8* and CD66b+ cell infiltrates; however, this may be due to CD66b being a more general granulocyte marker rather than neutrophil specific. We hypothesise that *CXCL8* produced by CAFs recruits neutrophils to the TME and fosters an immunosuppressive pro‐TME. Combined treatment of *CXCL8*/CXCR2 inhibition with a standard‐of‐care chemotherapy represents an exciting approach to investigate in preclinical models of CRC.

The patients with high *CXCL8* expression in cohort 1 observed a higher frequency of mutations in the *CREBBP* gene. Mutation of *CREBBP* has been previously linked to worse prognosis in solid tumour types including head and neck cancer and CRC. *CREBBP* is an important signalling molecule involved in the regulation of various immune cell populations. It is important for IL10 production, regulatory T cell functions, or can conversely crosstalk with NF‐κB to promote transcription of pro‐inflammatory processes. Overactivation of signalling via mutation is likely linked to an immunomodulatory interaction with *CXCL8*.

Another hallmark of cancer promoted by *CXCL8* secretion is metastases and here we showed that *CXCL8* mRNA expression was enriched in CRC patients with stage IV disease. Previous literature has implicated *CXCL8* in promoting metastasis in pancreatic cancer, ovarian cancer, and CRC. In CRC cell lines, *CXCL8* drives EMT via a PI3K/AKT/NF‐κB axis [25]. In mouse models of pancreatic ductal carcinoma, CXCR2 inhibition resulted in reduced metastases and improved survival [26]. In this study, CXCR2+ cells were enriched in stroma‐dense primary and secondary tumours in the second cohort of matched colorectal primary tumours and liver metastases. Further work is required to determine if *CXCL8*/CXCR2 are responsible for driving this unfavourable stroma‐rich phenotype.

Limitations of this study include a lack of mechanistic work and future experiments should include *in vitro*/*in vivo* experiments to determine the effect of *CXCL8* ablation in fibroblasts cocultured with CRC cell lines/organoids and preliminary pathway inhibition to determine the therapeutic potential of drugs which target *CXCL8*/CXCR2 in right‐sided colon cancer models and in the metastatic setting. Multiplex immunofluorescence staining should be employed to explore the influence of the spatial distribution of *CXCL8* on survival outcomes in more detail, for example by assessing the co‐localisation of α‐SMA and *CXCL8*. To conform to REMARK guidelines, future experiments should also include the utilisation of a validation cohort to confirm the findings of this study. The cohorts utilised in this study lacked granularity of treatment data, so it was not possible to correlate *CXCL8* expression with response to chemotherapy/chemoradiotherapy type or duration. A validation cohort with full treatment data should be sought for future work. We were unable to identify an antibody of sufficient specificity and quality to detect *CXCL8* at the protein level to confirm translation from mRNA, which represents another limitation of the current study.

To conclude, this study has demonstrated a clear role for *CXCL8* in the CRC setting. From data acquired thus far, we hypothesise that *CXCL8* is involved in promoting a stroma‐rich microenvironment which aids tumour immune evasion and EMT, and that targeting this pathway could be therapeutically beneficial in a subset of patients with right‐sided tumours.

## Author contributions statement

KAFP performed IHC, scored RNAscope and IHC, performed data analysis and wrote the manuscript. JAQ generated experimental data and edited the manuscript. CN performed RNAscope and edited the manuscript. JI performed IHC for the immune cells and edited the manuscript. HCvW assessed tumours for tumour budding and was involved in pathological assessment. DC, SR and the GPOL Group performed mutational analysis. JH constructed TMAs, performed MMR analysis and edited the manuscript. NNM is a consultant pathologist who provided all pathological training and double scoring, ensured quality of pathological assessment and edited the manuscript. CSDR and PGH are consultant colorectal surgeons who established clinical cohorts and built clinical databases. DCM analysed the data and edited the manuscript. JHP and AKR performed TSP and KM assessment for the Glasgow Microenvironment Score. CWS performed data analysis and was involved in writing and editing of the manuscript. JE conceived the study, is grant PI and was involved in pathological assessment, data analysis and editing of the manuscript.

## Supporting information


**Figure S1.** Patient inclusion criteria
**Figure S2.** The prognostic effect of high stromal *CXCL8* expression shows a similar trend in both pMMR and dMMR disease
**Figure S3.** Intra‐tumour stromal invasion
**Table S1.** Univariate and multivariate survival analyses

## Data Availability

Data are held in Greater Glasgow and Clyde Safe Haven (Safe Haven number: GSH/18/ON007).
