# Supplementary material for: CXCL8 expression is associated with advanced stage, right sidedness, and distinct histological features of colorectal cancer
Source: J Pathol Clin Res. 2022 Jul 25;8(6):509–20. doi: 10.1002/cjp2.290 (PMC9535100; doi:10.1002/cjp2.290)
Supplement: Supplementary file 1 — Figure S1. Patient inclusion criteria Figure S2. The prognostic effect of high stromal CXCL8 expression shows a similar trend in both pMMR and dMMR disease Figure S3. Intra‐tumour stromal invasion Table S1. Univariate and multivariate survival analyses [file CJP2-8-509-s001.pdf]

***CXCL8* expression is associated with advanced stage, right sidedness, and distinct histological features of colorectal cancer**

KAF Pennel *et al*. *J Pathol Clin Res* https://doi.org/10.1002/cjp2.290

Supplementary Figures S1 – S3

Supplementary Table S1

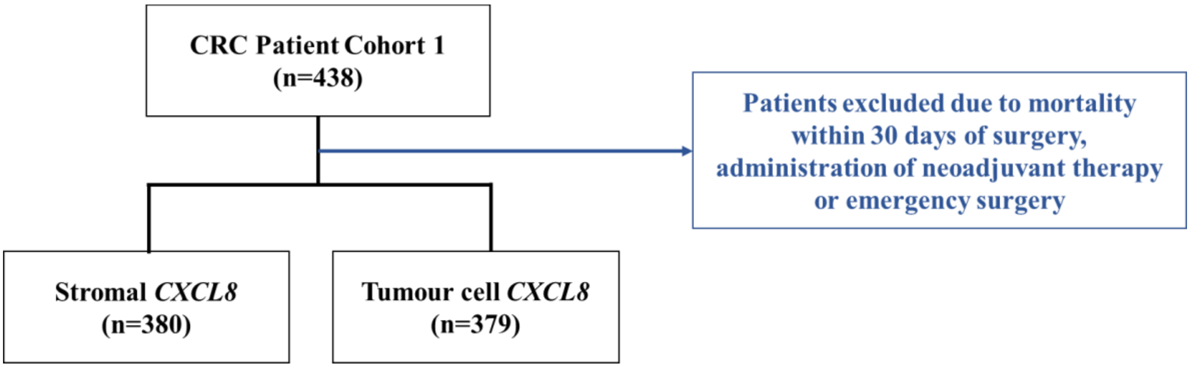

Figure S1. Patient inclusion criteria. Consort diagram detailing the number of patients included in final analysis of stromal and tumour *CXCL8* expression.

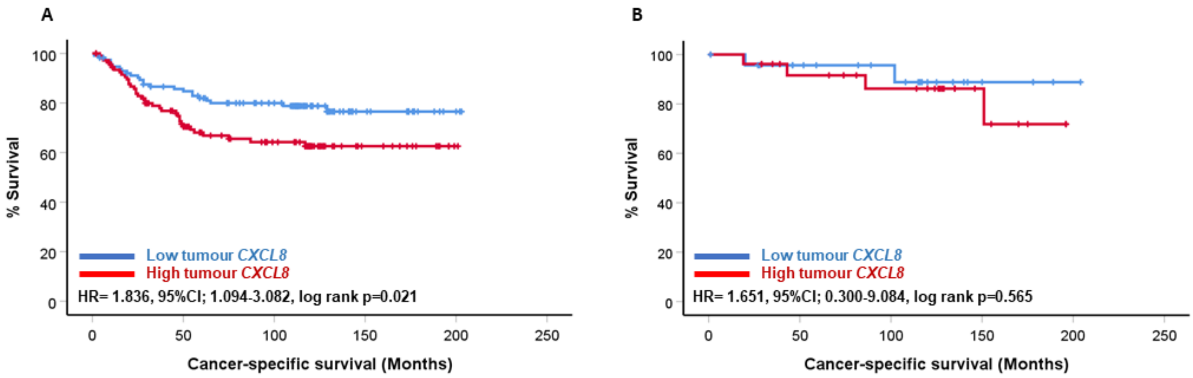

Figure S2. The prognostic effect of high stromal *CXCL8* expression shows a similar trend in both pMMR and dMMR disease. Kaplan Meier survival analysis of stromal CXCL8 expression in (A) the MMR proficient cases and (B) the MMR deficient cases.

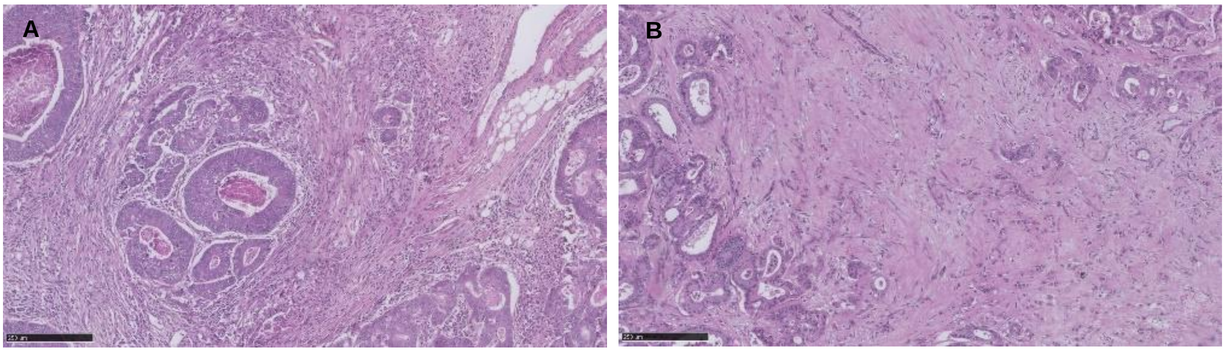

Figure S3. Intra-tumour stromal invasion. Representative images of high stromal invasion in (A) a primary and (B) a metastatic tumour.

Table S1. Univariate and multivariate survival analysis. Table showing results from Cox regression survival analysis when variables were assessed individually and then subsequent multivariate analysis including only the variables significant at the univariate level.

	**Univariate analysis**		**Multivariate analysis**	
**Clinical Characteristic**	HR (95%CI)	p	HR (95%CI)	p
Age (≤65/>65)	1.193 (0.882-1.615)	0.252	-	-
Sex (male/female)	1.163 (0.878-1.540)	0.292	-	-
Tumour site (colon/rectum)	1.128 (0.834-1.525)	0.434	-	-
T-Stage (1/2/3/4)	1.848 (1.498-2.281)	<0.001	2.437 (1.571-3.782)	<0.001
N-Stage (0/1/2)	2.168 (1.813-2.592)	<0.001	1.558 (1.089-2.231)	0.015
M-Stage (0/1)	7.363 (3.985- 13.605)	0.017	2.933 (0.961-8.950)	0.059
Ki67 Index (≤30%/>30%)	0.521 (0.389-0.696)	<0.001	0.633 (0.368-1.019)	0.097
**MSI status (MSI-d/MSI-h)**	0.659 (0.449-0.967)	0.033	0.515 (0.251-1.057)	0.070
**Tumour stroma % (≤50%/>50%)**	2.060 (1.525-2.782)	<0.001	1.764 (1.091-3.052)	0.043
**Klintrup-Mäkinen grade (0-1/2-3)**	0.367 (0.256-0.527)	<0.001	0.282 (0.119-0.668)	0.004
**Tumour budding (low/high)**	1.383(1.014-1.887)	0.040	0.993 (0.569-1.735)	0.981
**Stromal *CXCL8* (low/high)**	1.679 (1.030-2.737)	0.038	1.366 (0.796-2.344)	0.071
